# High-dimensional investigation of the cerebrospinal fluid to explore and monitor CNS immune responses

**DOI:** 10.1186/s13073-022-01097-9

**Published:** 2022-08-17

**Authors:** Michael Heming, Anna-Lena Börsch, Heinz Wiendl, Gerd Meyer zu Hörste

**Affiliations:** Department of Neurology with Institute of Translational Neurology, University Hospital Münster, Münster, Germany

**Keywords:** Cerebrospinal fluid, Single-cell transcriptomics, Multiple sclerosis, Alzheimer’s disease, Parkinson’s disease, COVID-19, Brain metastases, Single-cell atlas

## Abstract

The cerebrospinal fluid (CSF) features a unique immune cell composition and is in constant contact with the brain borders, thus permitting insights into the brain to diagnose and monitor diseases. Recently, the meninges, which are filled with CSF, were identified as a neuroimmunological interface, highlighting the potential of exploring central nervous system (CNS) immunity by studying CNS border compartments. Here, we summarize how single-cell transcriptomics of such border compartments advance our understanding of neurological diseases, the challenges that remain, and what opportunities novel multi-omic methods offer. Single-cell transcriptomics studies have detected cytotoxic CD4^+^ T cells and clonally expanded T and B cells in the CSF in the autoimmune disease multiple sclerosis; clonally expanded pathogenic CD8^+^ T cells were found in the CSF and in the brain adjacent to β-amyloid plaques of dementia patients; in patients with brain metastases, CD8^+^ T cell clonotypes were shared between the brain parenchyma and the CSF and persisted after therapy. We also outline how novel multi-omic approaches permit the simultaneous measurements of gene expression, chromatin accessibility, and protein in the same cells, which remain to be explored in the CSF. This calls for multicenter initiatives to create single-cell atlases, posing challenges in integrating patients and modalities across centers. While high-dimensional analyses of CSF cells are challenging, they hold potential for personalized medicine by better resolving heterogeneous diseases and stratifying patients.

## Background

The cerebrospinal fluid (CSF) is a clear liquid, an ultrafiltrate of the blood, ensheathing the central nervous system (CNS). Produced by the choroid plexus (CP) and ependymal cells in the brain ventricles, the CSF circulates to the subarachnoid space until being drained into the dural venous system through arachnoid villi [1]. Alternatively, the CSF flows directly into meningeal lymphatics [2, 3] or along cranial and spinal nerves into adjacent lymphatics [1]. There is also likely CSF influx into the brain parenchyma through periarterial spaces and efflux via paravenous spaces back into the subarachnoid space [4].

When diagnosing brain diseases, neurologists have to balance the potential benefit of correct and timely diagnosis against the risk of potentially invasive diagnostic procedures. A brain biopsy is often considered the “last resort” due to its potentially fatal complications (mortality 1–3.5% [5–7]). Studying CNS tissue is thus hampered by limited sample accessibility of human CNS tissue, which is even more rarely available for research purposes. This limits options for studying immune cells surrounding the human CNS to imaging approaches, rare biopsy/autopsy material, or analyzing CSF. Since the CSF is in constant contact with the brain borders, it facilitates insights into the brain and can be used to monitor CNS immune responses without the need for an invasive brain biopsy. In this review, we discuss how technological advances in single-cell sequencing have been used to understand CNS immunity in a variety of complex neurological diseases by characterizing CSF cells with unprecedented resolution.

### CSF—a unique immune tissue

The CSF is a unique biomaterial both from a biological and medical point of view. The CSF bathes the brain, thus decreasing the weight of the brain by buoyancy from 1500 to 50 g [8]. Moreover, it provides trophic support for the CNS and controls lymphocyte and antigen shuttling from and towards the CNS parenchyma [3]. The volume of the CSF ranges between 125 and 150 ml in humans and a constant CSF production of approximately 25 ml/h results in complete CSF exchange of approximately four times per day [8]. Although the non-cellular fraction of the CSF is essentially a size-dependent ultrafiltrate of the blood, cells residing in the CSF are far from representing a mere “flow-over” of cells from the blood. Except for occasional ependymal debris or tumor cells, the cells found in the CSF are exclusively of hematopoietic origin, and therefore, the term CSF cells is generally and henceforth used synonymously to CSF leukocytes. However, CSF leukocytes are quantitatively and qualitatively disparate from blood leukocytes. The leukocyte concentration in the CSF is approximately 1000-fold lower than in the blood [9]. CD4^+^ T cells, in particular, activated central memory CD4^+^ T cells [10], dominate the healthy CSF, while myeloid-lineage cell numbers are low compared to blood [9, 10]. Using an unbiased single-cell transcriptomic approach, we recently characterized this CSF-specific cell composition with high resolution, identifying an increase of myeloid and plasmacytoid dendritic cells, CD4^+^ T cells, and regulatory T cells compared to blood. Inversely, B cells, plasma cells, granulocytes, NK cells, and monocyte subsets are reduced compared to blood [11]. In fact, CSF contains a monocyte subpopulation with a microglia-like phenotype [12–14], which is almost exclusive to the CSF [11]. This CSF microglia-like population was found to originate from the bone marrow in a single bone marrow transplant recipient patient [15], in contrast to resident microglia of the CNS, which derive from the yolk sac [16, 17]. Transcriptionally CSF T cells show enhanced expression of transcripts associated with migration (*CD99*), interaction with antigen-presenting cells (APC) (*CD83*, *CD84*) and chemokines (*CXCL16*, *CXCR5*), while genes associated with naive cell state (*SELL*), cytokine response (*IL2RG*), and integrins (*ITGAL*, *VLA4*) are reduced in CSF T cells compared to blood T cells [11]. The healthy CSF also contains clonally expanded T cells, which are presumably shared between blood and CSF [18]. In summary, the leukocyte composition and phenotype of CSF cells are distinct from the blood, indicating that the leukocyte composition and phenotype are tightly controlled by site-specific mechanisms [19].

### CSF—an important diagnostic tool

To gain access to the CSF, a lumbar puncture (LP) can be performed quickly and at a relatively low risk [20] compared to a much more invasive brain biopsy. While the first reported LPs were performed already in the 19th century by Quincke and Essex [21], this technique still constitutes an essential diagnostic procedure in clinical neurology in most countries. However, in clinical neurology in most centers worldwide, CSF analysis remains limited to classification of CSF cells into basic hematopoietic lineages, quantification of protein, lactate, and glucose, calculating the CSF/serum quotient of albumin and immunoglobulins and testing for the synthesis of oligoclonal immunoglobulins in the CSF. Despite modern high-resolution imaging techniques, CSF analysis remains indispensable in clinical neurology in the diagnosis of common neurologic diseases, including meningitis, encephalitis leptomeningeal metastases, and small subarachnoid hemorrhage [22]. In multiple sclerosis (MS), the most common neuroinflammatory disorder of the CNS, the synthesis of oligoclonal immunoglobulins in the CSF has been included in the latest revision of the MS diagnostic criteria [23]. This shows that CSF analysis remains an important tool in modern clinical neurology. However, we believe that the potential of the CSF could be exploited to a far greater extent by performing higher-dimensional analyses of the CSF.

### Meninges and the CSF—neuroimmunological interface and gateway to the brain

**Fig. 1:**
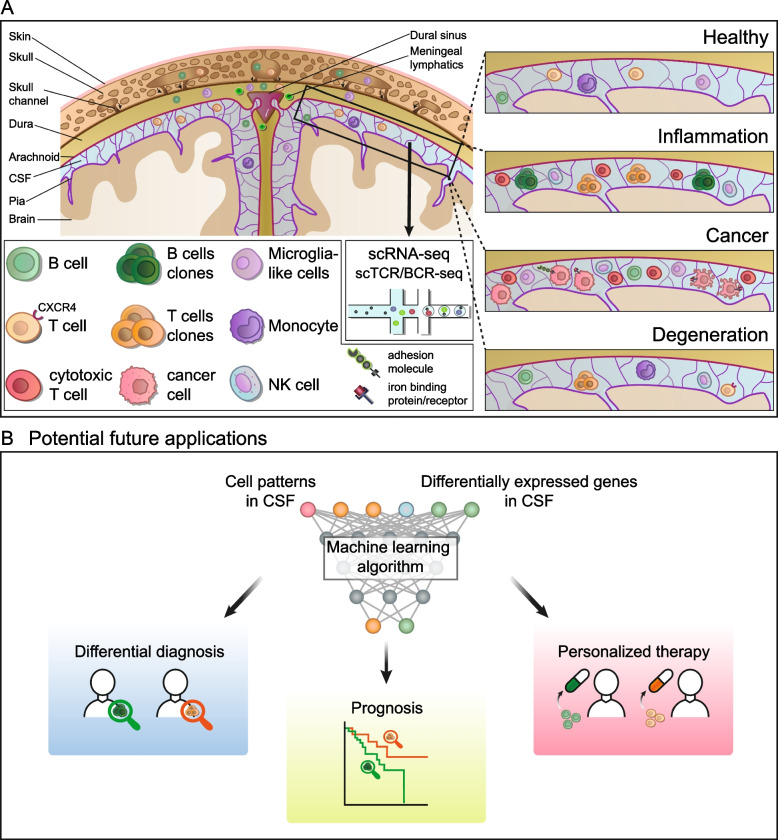
High-dimensional analysis of the diseased CSF. **A** Schematic illustration of the brain parenchyma, the cerebrospinal fluid (CSF), the meninges, and the skull. Immune cells can migrate from the skull bone marrow through skull channels to the dura layer of the meninges, where they accumulate in the vicinity of dural sinuses. Main findings of single-cell transcriptomics studies of the CSF are visualized in the upper right, including cytotoxic T cells and clonal expansion of B and T cells in inflammatory diseases, cancer cells with iron-binding protein/protein and adhesion molecules in tumors, and clonally expanded T cell in neurodegenerative disorders (see Table 1 and main text for details). **B** Potential future applications. We envision that cell patterns and the transcriptomic profile of CSF single-cell analysis will be utilized in the future to train machine learning algorithms to predict the clinical outcome, support differential diagnosis and permit a personalized therapy.

Improved understanding of the CSF is immediately interconnected with a better understanding of the meninges since these fibrous membranes are filled with CSF and wrap the brain (Fig. 1). The meninges consist of three layers: the outer dura mater, the arachnoid mater, and the pia mater with the CSF located in the subarachnoid space. While traditionally the meninges were considered as inert fibrous membranes solely providing mechanical protection to the brain, several recent studies redefined the meninges as a pivotal site of immune cell residence [24]. In fact, meningeal immune cells likely provide immunological protection against infections. In a recent study, IgA^+^ plasma cells were detected adjacent to dural venous sinuses, protecting the brain from infection by entrapping pathogens [25]. This provides evidence that meningeal immune cells are vital to maintaining CNS health. Shortly afterwards, we and others identified the meninges, and specifically, the dura, as an unexpected site of B cells and B cell progenitors that are usually not found outside the bone marrow [26–28]. The meninges also host myeloid cells that do not originate from the blood [29]. This indicates that meningeal immunity is not only important, but also developmentally and phenotypically unique. Both myeloid and B cell lineage might either develop directly in the meninges, mainly the dura [26], or derive from the skull bone marrow and migrate to the meninges through specialized skull channels [27, 29] (Fig. 1). The verdict is still out on whether the influx from skull bone marrow vs. local development model is correct and both hypotheses are not necessarily mutually exclusive. Collectively, these studies identified CNS border compartments, particularly meninges, as a novel neuroimmunological interface with various immune cell populations and thus need to be viewed in conjunction with CSF immune cells.

Besides studying immune cells in CNS-associated compartments, investigating the flux of CNS antigens is critical in order to better diagnose and treat neurological diseases because autoimmunity against CNS antigens can occur in multiple brain diseases. CNS antigen efflux occurs from the brain parenchyma via paravenous spaces through the CSF [4] to the peri-sinusoidal dura, where they are presented to patrolling T cells by dural antigen-presenting cells (APC) [3]. Dural sinuses thus may orchestrate immune surveillance of the brain [3]. CSF also enters the skull bone marrow, where it instructs local hematopoiesis [30]. These concepts have been translated to murine disease models. Autoreactive T cells that recognize myelin oligodendrocyte glycoprotein (MOG) are negatively selected in the meninges [28] and neuroinflammation induces an immune regulatory niche in local meningeal lymphatic vasculature [31]. Disruption of meningeal lymphatics diminishes MS [32], but deteriorates Alzheimer’s disease (AD) [33] in mouse models. Meningeal lymphatics exist also in humans as recent visualizations illustrate [34, 35]. In essence, a model emerges, in which the CSF, the meninges, and the overlying skull bone marrow might integrate into interconnected immunological barrier sites for the brain with protective functions in homeostasis and with negative effects when locally supporting autoimmunity [36]. Many aspects of this novel and partly hypothetical concept of a ‘peri-brain immune system’ are beginning to be resolved by using single-cell technologies.

### Diseased CSF in the single-cell transcriptomics age—a new era

Single-cell RNA sequencing (scRNA-seq), method of the year in 2013 [37], has revolutionized many scientific fields, including neurology and neuroscience by enabling the dissection of cellular heterogeneity within complex tissues at an unprecedented resolution [38, 39]. There has been a recent ‘boom’ in translational scRNA-seq studies with relevance to clinical neurology [39, 40]. This is mainly due to a dramatic reduction of sequencing costs in combination with a commercially available microfluidics-based approach [41, 42], which allows the preparation of thousands of cells in one sample by using cell barcodes and unique molecular identifiers (UMIs), thus reducing cost and work time significantly. Due to its low input and immune cell heterogeneity, CSF is well suited for analysis by scRNA-seq [39]. In contrast to flow cytometry, scRNA-seq permits a hypothesis-free cell type identification with thousands of genes detected instead of a limited panel of antibodies of predefined markers. To establish an overview of scRNA-seq CSF studies, we provide a list of published single-cell transcriptomics studies of the CSF in Table 1. Main findings are depicted in Fig. 1 and we discuss the results and the implications for a more comprehensive and unbiased characterization of CNS immunity in complex neurological disorders in further detail in the following sections.

**Table 1:** Overview of scRNA-seq CSF studies

Category	Disease	Samples	Main findings	Reference
**Inflammation**	MS AIE	MS-affected twins: 4 CSF, 4 PBMC “healthy” MS-twins: 8 CSF, 8 PBMC AIE: 2 CSF, 2 PBMC Controls: 2 CSF, 2 PBMC	Clonally expanded CD8^+^ T cells in the CSF of MS and SCNI, plasmablasts in the CSF of MS, SCNI, and AIE	[43]
	MS	MS: 6 CSF, 6 PBMC Controls: 6 CSF, 6 PBMC	B/plasma cells, NK, CD8^+^/CD4^+^ T cells, TFH in the CSF of MS, cytotoxic CD4^+^ T_EMRA_ cells in the CSF of MS	[11]
	MS OND	MS: 12 CSF, 12 PBMC OND: 1 CSF, 1 PBMC Control: 3 CSF, 3 PBMC	Clonally expanded B cells associated with inflammation and blood-brain in the CSF of MS	[14]
	MS	MS: 5 CSF, 5 PBMC Controls: 6 CSF, 6 PBMC	Activated and cytotoxic phenotype of clonally expanded T cells in the CSF of MS	[18]
	IgG4-RD	IgG4-RD: 1 CSF	CD8^+^ and CD4^+^ T cells, B cells in the CSF of IgG4-RD	[97]
	MS ONID	MS: 19 CSF ONID: 15 CSF Controls: 2 CSF	Plasma cells and T cells in the CSF of MS increase of myeloid cells and Reduction of B and T cells in anti-CD20 treated MS	[15]
**Degeneration**	AD MCI PD	AD: 7 CSF MCI: 5 CSF PD: 8 CSF Controls: 14 CSF	Clonally expanded CD8^+^ T_EMRA_ cells in the CSF of AD	[50]
	AD MCI PD	AD: 4 CSF MCI: 7 CSF PD: 7 CSF Controls: 8 CSF	TCRs similarity in the CSF of AD and MCI	[93]
	LBD	LBD: 11 CSF Controls: 11 CSF	CXCR-expressing CD4^+^ T cells in the CSF of LBD	[52]
**Cancer**	BRCA NSCLC	BRCA: 3 CSF NSCL: 5 CSF	Iron-binding protein and its receptor expressed by cancer cells in the CSF	[54]
	LUAD	LUAD: 5 CSF Controls: 3 CSF	Increased transcripts of cell adhesion and metabolic pathways in circulating tumor cells in the CSF	[55]
	BRCA LUAD ESCA SCLC SKCM	BRCA: 1 CSF, 3 tumors LUAD: 4 CSF, 3 tumors ESCA: 2 CSF, 1 tumor SCLC: 1 tumor SKCM: 1 CSF, 1 tumor	Identical TCR clones in the CSF and the brain in patients with brain metastasis	[57]
	PCNSL	PCNSL: 8 CSF	Intratumor heterogeneity in the CSF of PCNSL	[59]
	Melanoma	Melanoma: 18 CSF, 22 tumor Control: 2 CSF	Increased dysfunctional T cells in the CSF from patients with leptomeningeal than with brain/skin metastases	[60]
**Infection**	HIV	HIV: 3 CSF, 2 PBMC Control: 2 CSF	Microglia-like cells in the CSF of HIV	[13]
	COVID-19 VE MS	Neuro-COVID: 8 CSF VE: 5 CSF MS: 4 CSF Controls: 5 CSF	Exhausted CD4^+^ T cells and differentiated monocytes in the CSF of Neuro-COVID, less pronounced interferon response in the CSF of Neuro-COVID compared to VE	[61]
	COVID-19	COVID-19: 5 CSF, 6 PBMC Control: 6 CSF	T cell activation, clonal T cell expansion, B cell enrichment, and anti-neuronal autoantibodies in the CSF of COVID-19	[62]

Please note that in some cases the number of patients is ambiguous because samples were excluded for certain analyses or immune repertoire data was only available for a subset of patients. Reanalyzed samples, which were previously published, were not taken into account

Abbreviations: *AD* Alzheimer’s disease, *AIE* autoimmune encephalitis, *BRCA* breast cancer, *CSF* cerebrospinal fluid, *ESCA* esophagus carcinoma, *IgG4-RD* IgG4-related disease, *LBD* Lewy body dementia, *LUAD* lung adenocarcinoma, *MS* multiple sclerosis, *MCI* mild cognitive impairment, *NSCLC* non-small cell lung cancer, *NMOSD* neuromyelitis optica spectrum disease, *OND* other neurological diseases, *ONID* other neuroinflammatory disorders, *PD* Parkinson’s disease, *SCLC* small cell lung cancer, *SCNI* subclinical neuroinflammation, *scRNA-seq* single-cell RNA sequencing, *scTCR/BCR-seq* single-cell T/B cell receptor sequencing, *SKCM* skin cutaneous melanoma, TEMRA, *VE* viral encephalitis

### Dissecting immune responses in complex neuroinflammatory diseases by single-cell transcriptomics of the CSF

The paradigmatic neuroinflammatory disease MS has been most extensively studied via single-cell transcriptomics of the CSF (Table 1). A study of MS-discordant monozygotic twins detected clonally expanded CD8^+^ T cells in MS, but also in MS twins with subclinical neuroinflammation and in two autoimmune encephalitis (AIE) patients [43]. Plasmablasts were also identified in MS patients and MS-twins with subclinical neuroinflammation, while absent in healthy patients [43]. The findings imply that immune cell alterations precede the clinical manifestation of MS. The disease may thus be detectable preclinically by analyzing CSF leukocytes, and pathological immune alterations in preclinical stages could provide a rationale for early immunomodulating treatment. Our group described an increased proportion of B, plasma, NK, CD8^+^, CD4^+^ T cells, and follicular T helper cells (TFH) in the CSF of MS patients [11]. Such TFH cells accordingly exacerbated two animal models of MS [11, 36]. One cluster of CD4^+^ T cells showed a cytotoxic transcriptional phenotype, which is enriched among the effector memory recently activated pool of CD4^+^ T cells (CD4^+^ T_EMRA_) cells [44]. We confirmed that such CD4^+^ T_EMRA_ cells expand in the CSF in MS [11]. A further scRNA-seq study also found clonally expanded B cells in the CSF of MS patients, that were transcriptionally associated with inflammation and blood-brain barrier breakdown, while no clonal expansion was observed in healthy patients [14]. At the same time, another group examined the CSF of MS patients and found an activated and cytotoxic phenotype of clonally expanded T cells [18]. However, the authors did not find an increase of expanded T cells in MS compared to healthy and no clonal overlap between MS patients [18], in contrast to Beltran et al. [43]. The authors argue that this could be explained by T cells that migrate from the CSF to demyelinated lesions [18]. Additionally, the deviating findings might be due to the inherent heterogeneity of MS [45] and the small sample sizes. A recent preprint generated a large scRNA-seq CSF dataset of neuroinflammatory, mostly MS, and control patients [15]. Next to a reduction of microglia-like cells and the well-known expansion of plasma cells and T cells in the CSF of MS patients, the authors observed a reduction of B and T cells and an increase of myeloid cells of ocrelizumab (anti-CD20) treated progressive MS patients versus therapy-naive relapsing-remitting MS patients [15]. The authors speculate that a *CD27* downregulation in plasma cells in ocrelizumab-treated MS patients might mediate the immunomodulatory effect of the therapy [15].

While these studies excel through their transcriptional resolution on a single-cell level, many studies lack methodical validation on the protein levels, although some [11, 14] provide flow cytometry verification. In addition, clinical validation cohorts would be preferable, but the technique yet remains prohibitively expensive. We find that the field would benefit from a meta-analytic integration of available single-cell datasets across tissues, centers, and neurological diseases to confirm or refute findings from individual studies with higher statistical power. Nonetheless, available data already demonstrate that high-dimensional scRNA-seq analyses of the CSF can successfully dissect complex neuroinflammatory diseases. Single-cell technologies will likely be extended to study the effect of different immunotherapies on CNS immunity and gain insights into the pathogenesis of progressive multiple sclerosis. Such in-depth understanding could facilitate individualized and targeted therapies in MS.

While it is not completely resolved if findings in the CSF fully reflect pathological processes in the CNS, several parallel findings in both tissues point into this direction. Clonally expanded CD8^+^ T cells are an important hallmark in the CSF of MS patients and are also the dominant infiltrating cell population in the CNS in MS [46, 47]. Additionally, CD8^+^ T cell clones can be shared between the brain parenchyma and the CSF [48]. Demyelinating lesions in MS commonly show meningeal infiltration of plasma cells [49] in line with their increase in CSF [11, 14, 43]. Collectively, CSF is likely a suitable surrogate tissue to study the immune cell infiltration of the meninges and the brain parenchyma in inflammatory disorders. Nonetheless, studies with paired samples from CSF, dura, and the CNS in MS would be desirable to substantiate this point.

### Single-cell transcriptomics of the CSF to advance understanding in neurodegenerative disorders and neuro-oncology

High-dimensional CSF analysis via scRNA-seq has also been successfully employed in neurodegenerative disorders, leptomeningeal tumor metastasis, and neurological infections (Table 1). In a recent study, scRNA-seq was combined with single-cell T cell receptor sequencing (scTCR-seq) to reveal clonally expanded CD8^+^ T_EMRA_ cells in the CSF of AD patients [50], a dementia associated with extracellular β-amyloid and intracellular tau protein deposits in the brain [51]. Intriguingly, CD8^+^ T cells were also detected in post-mortem brains of AD patients, especially adjacent to β-amyloid plaques, hippocampi, and nearby leptomeninges [50], indicating that pathogenic CD8^+^ T cells enter the brain via the meninges and the CSF and could play a role in the pathophysiology of AD. In a further study, utilizing single-cell transcriptomics of the CSF, the authors detected increased expression of *CXCR4* in CD4^+^ T cells in patients with Lewy body dementia [52], a dementia characterized by α-synuclein deposits in the brain [53]. Intriguingly, the CSF concentration of CXCL12, ligand of CXCR4, correlated with neuroaxonal damage [52]. Collectively, these novel findings corroborate a decisive role of the immune system in neurodegenerative disorders, which are classically not thought to be immune disorders. Since T_EMRA_ cells were expanded in AD, their presence in the CSF could support clinicians in the differential diagnosis of dementia in addition to classical neurodegenerative markers (β-amyloid and tau protein). These findings might then pave the way to numerous therapeutic approaches. This is especially relevant because therapeutic options to treat dementia are still limited and therapies targeting specific immune cells are widely used in neuroinflammatory diseases.

By investigating the CSF of patients with leptomeningeal metastases using single-cell transcriptomics, the authors found that cancer cells, but not macrophages, express an iron-binding protein and its receptor [54]. Iron is sparse in the CSF and the iron-binding protein promotes cancer cell growth in the leptomeninges in mice [54]. The cancer cells might thus outcompete macrophages for sparse iron, resulting in a survival advantage [54]. The study serves as a good example of how high-resolution analysis of single CSF cells can pinpoint specific molecules in a complex disease and tissue. In lung cancer, circulating tumor cells in the CSF were deeply characterized with scRNA-seq, which simultaneously resolves cellular composition and expression. The authors detected transcripts of metabolic pathways and cell adhesions, which are required for survival and metastasis of tumor cells [55]. This included complement protein C3, which is necessary for cancer growth in the leptomeninges [56]. In a case of cancer of unknown primary, the authors identified markers of the CSF tumor cells, which characterized the tumor deeper than conventional immunohistochemistry results and narrowed down its origin [55]. In a different study, patients with brain metastasis of different origins showed identical cytotoxic CD8^+^ T cell clonotypes in the brain parenchyma and the CSF and selected TCR clones persisted after the therapy [57]. This indicates that the CSF can be used to monitor clonal T cell evolution and potentially guide the therapy in brain metastasis by using identical T cell clones in the CSF to design cell therapies. Furthermore, the findings provide evidence that the CSF is a suitable tissue to study infiltrating immune cells of the brain parenchyma in brain metastasis. This concept is well known in oncology as “liquid biopsy” and not only holds potential to answer scientific questions because of its better accessibility, but also to track resistant clones and detect early relapse in clinical oncology [58]. In primary CNS lymphoma, the analysis of CSF tumor cells displayed substantial heterogeneity within patients [59]. Dissecting the tumor heterogeneity is important because it lays the foundation for identifying malignant therapy-resistant subclones and eventually designing therapeutic protocols targeting resistant cells [58]. In a recent study of melanoma metastases, the authors discovered more dysfunctional T cell proportions in the CSF from patients with leptomeningeal than with brain or skin metastases [60]. Moreover, the CSF of an exceptional therapy responder featured a distinct cellular composition compared to poor responders and showed an increase of functional effector memory T cells after treatment [60]. These findings illustrate how scRNA-seq CSF analysis can be used to better understand treatment response and potentially guide treatment decisions in the future. Collectively, single-cell transcriptomics of CSF cells thus holds diagnostic and prognostic potential in oncology with potential therapeutic implications.

### Single-cell transcriptomics of CSF to study sequelae of COVID-19—the new pandemic?

In the current COVID-19 pandemic, we and others leveraged scRNA-seq of CSF to investigate the CNS immune response of COVID-19 patients [61, 62]. Since the neurological involvement of COVID-19 is poorly understood and blood does not represent CNS inflammation well, a high-dimensional analysis of the CSF of Neuro-COVID patients was a suitable approach. We observed an expansion of exhausted CD4^+^ T cells and dedifferentiated monocytes in acute COVID-19 patients with neurological manifestations in the CSF, termed Neuro-COVID [61]. In comparison to viral encephalitis, Neuro-COVID patients showed a less pronounced interferon response that was curtailed in severely affected Neuro-COVID patients [61]. Using scTCR-seq we found evidence for a broad clonal T cell expansion in severe Neuro-COVID patients [61]. Another group reported transcriptional T cell activation, clonal T cell expansion, B cell enrichment, and anti-neuronal autoantibodies in the CSF of Neuro-COVID patients [62]. The findings suggest immune-mediated mechanisms causing damage to the nervous system and provide evidence for investigating immunomodulating treatments in Neuro-COVID. Single-cell transcriptomics of the CSF is therefore suited to dissect the immune response in infectious diseases affecting the CSF. It might also be a powerful tool to investigate long-term post-COVID-19 neurological sequelae.

### Disease monitoring and clinical management of patients based on high-dimensional CSF analysis

While a detailed knowledge about the cellular and transcriptional landscape of the CSF is of great importance, the ultimate aim of translational research should be to improve patient care. In the field of oncology, recent studies utilized single-cell transcriptomics to predict the clinical outcome. In gastric adenocarcinoma, tumor cells were classified into two subtypes based on their single-cell profile and a strong association with patient survival was observed [63]. The authors of a different study applied scRNA-seq and single-cell protein activity in renal carcinoma and detected a C1Q^+^TREM2^+^APOE^+^ macrophage subpopulation, which was significantly correlated with tumor relapse [64]. Additionally, single-cell transcriptomics was utilized to predict the therapy response in melanoma patients. A CD8^+^ T cell subpopulation with high levels of oxidative phosphorylation was identified that distinguished immune checkpoint inhibitor responders from non-responders [65]. Similar approaches could be translated to neurology, supporting clinicians in challenging decisions. This includes distinguishing MS from other neuroinflammatory disorders (ONIDs) and predicting the course of illness at an early stage (Fig. 1). Moreover, we envision that findings from high-dimensional CSF analysis could be utilized to train machine learning algorithms to predict the response to individual treatments (Fig. 1). Predictive models bear the advantage that they are capable of using multiple information, such as several abundant cell types and multiple differentially expressed genes, instead of focussing on a single parameter, as it is currently common practice in clinical medicine. Because of the diversity of available immunotherapies in MS [66], tailoring treatments to individual MS patients is especially important. In neurodegenerative disorders like AD, the detection of CD8^+^ T_EMRA_ cells in the CSF and the brain parenchyma [50] substantiate existing evidence that immunomodulating therapies might be effective in AD [67, 68]. With limited therapeutic options in AD and trials mostly focussing on β-amyloid, tau, and microglia modulation, T cells could represent an additional promising therapeutic target, illustrating how understanding the CSF can extend therapeutic options in the future.

### High-dimensional CSF analysis in animal models

The diagnostic value and widespread collection of CSF in human patients contrast sharply with the surprisingly limited information available from animal studies. This might be due to technical challenges in obtaining CSF from the most widely used laboratory species: mice [69]. In addition, the maximum CSF volume collectable from mice is limited to 10–15 μL [69]. Notably, this limited volume has been utilized to study CSF clearance in mice [70]. Moreover, several studies investigated the CSF in rodent AD models [71–73]. Using transgenic mice [71, 72] and rats [73], these studies could provide mechanistic insights, e.g. that β-amyloid pathology causes a biomarker profile observed in AD, even in the absence of tau aggregation and neuronal losses [73]. However, only one study performed a high-dimensional characterization of CSF cells [26]. In this study, we used rats because they provide higher CSF volumes of up to 100–120 μL [74] and consequently higher cell numbers. We still had to pool CSF from 20 rats to achieve a sufficient amount of cells for sequencing [26]. By simultaneously analyzing leukocytes in the brain parenchyma, dura mater, choroid plexus, pia mater, arachnoid, and the CSF, we found unique compositions in each compartment with surprisingly large proportions of B cells in the dura [11]. While animal models additionally often do not translate to humans [75], they permit performing more rigorous mechanistic studies and collecting CNS-associated border compartments more comprehensively. We therefore consider high-dimensional CSF studies in animals a valuable tool, whose potential has not been exploited yet.

### Integrative single-cell analysis in the CSF—opportunities and challenges

Recent advances in single-cell transcriptomics have led to great opportunities, but also major challenges. It is possible to investigate the transcriptome of thousands or even millions of cells [76] between different diseases, timepoints, and tissues to answer scientific and clinical relevant questions that could not have been addressed previously. However, there are several issues related to single-cell transcriptomics summarized in Table 2. Due to a low starting amount, transcripts can be missed during reverse transcription, which leads to “dropout” events, the presence of a gene at moderate/high expression in one cell but absence in another cell [77]. Consequently, the gene coverage of most scRNA-seq platforms is limited so that genes that are expressed at lower levels but are biologically important can be missed. Complex distributions of transcript abundances have led to an ongoing discussion about the optimal normalization method [78]. Moreover, there are around 170 integration tools available for scRNA-seq [79]. The most popular integration tools have recently been benchmarked [80] to assess their performance in removing batch effects, unwanted technical variation, while conserving biological variation. Nonetheless, it remains a challenge to identify the most appropriate tool and settings as each dataset requires individual settings. While this problem has already been tackled in integrative scRNA-seq studies of CSF and blood [81, 82], it will become even more challenging in the future with atlas initiatives, larger numbers of samples, and multi-omics approaches.

**Table 2:** Advantages and drawbacks of scRNA-seq of the CSF

Advantages	Drawbacks
Hypothesis-free in-depth characterization of cell populations [41]	Due to a low starting amount, transcripts can be missed during transverse transcription (“dropouts”), leading to a limited gene coverage [77]
Detection of novel disease- and cell-type-specific biomarkers	False positive and false negative DE genes can lead to false discoveries [98]
Can be combined with published CSF datasets to increase statistical power or non-CSF datasets to compare cell abundances or phenotypes between compartments, which improves the reproducibility across studies	Batch effect can be misinterpreted as novel biological findings while correction of batch effects entails the risk of removing biological variation [80, 99]
Wide range of analyses possible with a plethora of computation tools [100]	Analyses remain mostly descriptive and cannot substitute mechanistic experiments [101]
Because of limited CSF cell counts, deep-sequencing of CSF cells is affordable	Number of total available cells by limited by low CSF cell counts, thus relative cell frequencies can be biased and rare cell populations might be completely missed
Increasingly multi-dimensional data collected simultaneously (proteome, transcriptome, epigenome)	Because of limited CSF cell counts, differential expression of rare cell populations between conditions can be unreliable [102]

While the available CSF studies employed scRNA-seq and scTCR-seq, multi-modal approaches have emerged in the last few years that offer a plethora of opportunities to investigate the CSF in new ways. Cellular index of transcriptomes and epitopes by sequencing (CITE-seq) [83] and RNA expression and protein sequencing (REAP-seq) [84] allow simultaneous mRNA and cell surface protein detection. In contrast to flow-cytometry-based approaches, these methods allow a much larger amount of antibodies and measure both modalities at the same time. Consequently, immune cell types can be distinguished more finely [85]. Despite their tremendous potential, we found that the cell loss associated with staining procedures limits the applicability of CITE-seq/REAP-seq in CSF cells, which are naturally limited in number. The single-cell assay for transposase-accessible chromatin by sequencing (scATAC-seq) permits epigenomic profiling, thus revealing gene regulatory programs, e.g. detecting a regulatory network that governs exhaustion in tumor-infiltrating T cells [86]. Several approaches have been developed in the last years that combine scRNA-seq and scATAC-seq [87, 88]. Very recently a method has been introduced that couples cell surface and intracellular proteins with scATAC-seq (ASAP-seq) [89] and even an approach that enables measuring gene expression, chromatin accessibility, and protein in the same cell (DOGMA-seq) [89]. At the same time, computational tools were developed that allow the integration of multi-omics data in a weighted analysis [90]. Such a joint weighted analysis enables one data modality to compensate for the weakness of another, resulting in a higher resolution of cellular heterogeneity and a more holistic understanding. For example, T cells often form a phenotypic gradient in scRNA-seq [11] and the combination with protein data enables a better separation of T cell states [90]. Since T cells dominate the CSF, we assume that CSF analysis will benefit from multi-omics approaches. The resulting higher resolution will dissect the immune cells in the CSF more precisely than current methods. We believe that the use of multi-omics in combination with higher sample numbers can lead to a refined disease subtype classification in complex neurological diseases, such as MS and inflammatory polyneuropathies.

### Towards future atlas initiatives in the CSF realm

The number of cells in single-cell datasets is steadily growing and single-cell atlases are being generated. Recently, a large reference atlas of over 200,000 well-annotated human peripheral blood mononuclear cells processed with CITE-seq was published [90]. The Tabula Sapiens is a single-cell transcriptomic atlas of nearly 500,000 annotated human cells from 24 tissues and organs [91]. The Human Cell Atlas bundles atlas initiatives with the aim to identify the molecular profile of every human cell type with scRNA-seq and single-cell multi-omics as key technologies [92]. Most single-cell transcriptomics datasets of the CSF are relatively small so far (Table 1). We believe that the generation of a large reference atlas of CSF cells will necessitate establishing multicenter collaborations, optimally preserving CSF cells across centers, integrating existing datasets, and making the resulting annotated datasets publicly available, including an interactive visualization. A protocol for the cryopreservation of CSF has recently been published, which showed high post-thaw viability [93]. However, cell loss in CSF cryopreservation is a major concern given the low cell concentration and the limited volume of CSF. On the other hand, cryopreserved samples are well suited for multiplexing, either via cell hashing [94] or natural genetic variations [95, 96], which considerably reduces batch effects costs and experimental work. Data integration is a major challenge, but lessons can be learned from the existing large consortia. CSF atlases should be integrated into preexistent multitissue atlases, such as the Tabula Sapiens, because this enables direct comparisons between cells of different tissues. We believe that further and larger single-cell transcriptomics studies of the CSF will be extremely valuable to better understand neuroimmunological responses and neurological diseases in general.

## Conclusions

While CSF analysis has played an important role in clinical neurology for decades, recent high-dimensional methods, such as single-cell transcriptomics, are capable of exploring the CSF at unprecedented resolution. The meninges, filled with the CSF, have thus been identified as a neuroimmunological interface. Single-cell transcriptomics studies of the CSF have dissected the immune response in complex neurological diseases, including inflammatory, degenerative, infectious, and oncological CNS disorders. Important next steps will be to increase the number of samples by multi-center collaborations and integrate multi-omics approaches. This requires improved cell preservation methods, which is currently still hampered by cell loss, particularly with low CSF cells count, and careful bioinformatic analysis to tackle batch effects between individuals, tissues, and modalities. We envision that high-dimensional techniques like single-cell transcriptomics will be increasingly applied in challenging differential diagnosis, individualized prognosis, and prediction of therapy response.

## Data Availability

Not applicable.

## Abbreviations

AD: Alzheimer’s disease
AIE: Autoimmune encephalitis
APC: Antigen-presenting cells
CNS: Central nervous system
CSF: Cerebrospinal fluid
LP: Lumbar puncture
MS: Multiple sclerosis
MOG: Myelin oligodendrocyte glycoprotein
ONID: Other neuroinflammatory disorders
scRNA-seq: Single-cell RNA sequencing
scTCR/BCR-seq: Single-cell T/B cell receptor sequencing
T_EMRA_: T effector memory cells expressing CD45RA
TFH: T follicular helper cells
